# A three protein signature fails to externally validate as a biomarker to predict surgical outcome in high-grade epithelial ovarian cancer

**DOI:** 10.1371/journal.pone.0281798

**Published:** 2023-03-23

**Authors:** Amy Hawarden, Marcus Price, Bryn Russell, Godfrey Wilson, Laura Farrelly, Andrew Embleton-Thirsk, Mahesh Parmar, Richard Edmondson

**Affiliations:** 1 Division of Cancer Sciences, Faculty of Biology, Medicine and Health, University of Manchester, St Mary’s Hospital, Manchester, United Kingdom; 2 Department of Obstetrics and Gynaecology, Manchester Academic Health Science Centre, St Mary’s Hospital, Central Manchester NHS Foundation Trust, Manchester Academic Health Science Centre, Level 5, Research, Manchester, United Kingdom; 3 Department of Histopathology, Manchester University NHS Foundation Trust, Manchester, United Kingdom; 4 CRUK and UCL Cancer Trials Centre, UCL, London, United Kingdom; 5 Institute of Clinical Trials and Methodology, UCL, London, United Kingdom; Public Library of Science, UNITED KINGDOM

## Abstract

**Introduction:**

For patients with advanced epithelial ovarian cancer, complete surgical cytoreduction remains the strongest predictor of outcome. However, identifying patients who are likely to benefit from such surgery remains elusive and to date few surgical outcome prediction tools have been validated. Here we attempted to externally validate a promising three protein signature, which had previously shown strong association with suboptimal surgical debulking (AUC 0.89, accuracy 92.8%), (Riester, M., et al., (2014)).

**Methods:**

238 high-grade epithelial ovarian cancer samples were collected from patients who participated in a large multicentre trial (ICON5). Samples were collected at the time of initial surgery and before randomisation. Surgical outcome data were collated from prospectively collected study records. Immunohistochemical scores were generated by two independent observers for the three proteins in the original signature (POSTN, CXCL14 and pSmad2/3). Predictive values were generated for individual and combination protein signatures.

**Results:**

When assessed individually, none of the proteins showed any evidence of predictive affinity for suboptimal surgical outcome in our cohort (AUC POSTN 0.55, pSmad 2/3 0.53, CXCL 14 0.62). The combined signature again showed poor predictive ability with an AUC 0.58.

**Conclusions:**

Despite showing original promise, when this protein signature is applied to a large external cohort, it is unable to accurately predict surgical outcomes. This could be attributed to overfitting of the original model, or differences in surgical practice between cohorts.

## Introduction

Epithelial ovarian cancer (EOC) accounts for an estimated 239,000 new cases and 152,000 deaths worldwide annually [1]. Survival outcomes remain poor with the five-year survival for all stages being just 35% [2]. The majority of EOCs present with advanced disease, reflecting disease spread outside of the pelvis [3].

Treatment for EOC combines surgical resection of disease, platinum-based chemotherapy and more recently individualised maintenance therapies including PARP inhibitors [4]. Complete cytoreduction (no visible remaining disease following surgery) is the over-riding goal of surgical treatment, with overall survival (OS) being compromised if this is not achieved [5, 6]. Surgery can occur first-line, as primary debulking surgery (PDS) or if disease is deemed unresectable at the time of diagnosis as a second line treatment following chemotherapy, termed interval debulking surgery (IDS) [7–9]. Complete debulking at the time of PDS may hold a slight survival advantage over complete debulking at the time of IDS [10, 11]. However, tools to predict outcome remain elusive.

Many surgical prediction models have been published in the literature. These models utilise a combination of many different data modalities, including patient demographics, biochemical factors, radiological factors, genomic factors, and diagnostic laparoscopy. To date, only laparoscopy has externally validated with enough success to be considered for clinical use [12] but has not been widely adopted, in part because it is an invasive surgical procedure with an associated morbidity. Failure to develop and validate biomarkers of surgical outcome has been identified as a major deficit to the management of patients with advanced ovarian cancer [13].

An ideal biomarker would be simple, non-invasive, carry no additional morbidity for the patient, and would have a high degree of accuracy. Such a candidate biomarker was proposed by Riester *et al*, in 2014 [14]. The proposed model used expression of three proteins in stage III and IV high grade epithelial ovarian cancer tumours to predict suboptimal surgical debulking status with an accuracy of 92.8% and an area under the Receiver Operating Characteristic curve (AUC) of 0.89 [14].

The model utilises immunohistochemistry, a method that is used in all specialist histopathology labs [15] and could be applied using image guided biopsies which are now the standard diagnostic material for advanced ovarian cancer. Immunohistochemistry is therefore readily available, fast and cost effective [16].

Here we describe the external validation of the three-protein signature, using an independent cohort of patients recruited in the ICON 5 trial [17].

## Methods

Samples were accrued prospectively from patients enrolled in the UK based MRC ICON5 clinical trial [17]. Patients with stage III or IV epithelial ovarian cancer were enrolled into ICON5 following primary cytoreductive surgery between 2001 and 2004, Table 1. Patients were all WHO performance status 0–2 and had sufficient bone marrow, kidney and neurological function to be considered for chemotherapy. Following enrolment into ICON 5, patients were randomised to one of five chemotherapy arms but critically for the current study randomisation took place following surgery and tissue collection. Surgical outcome was therefore independent of allocated treatment arm. All clinical data were recorded prospectively as part of the clinical trial protocol but were kept blinded to the laboratory team until scoring and analysis had been completed. Included tumours had ≥ 3 available slides to allow for staining for each of the three proteins and for negative controls.

**Table 1:** Clinical characteristics of patients in original test set and current validation set.

	Test dataset	Validation dataset	p value
	n = 178	n = 238	
Patient age	unknown	unknown	
Stage at diagnosis n (%)			
III	142 (80%)	204 (85.7%)	
IV	36 (20%)	34 (14.3%)	
Primary debulking surgery n (%)	100%	85%	
Suboptimal debulking rates n (%)	43 (24%)	112 (47%)	p <0.0001
Dates surgeries performed	1993–2009	2001–2004	
Age of samples at time of IHC	4–20 years	15–18 years	
Single or multicentre	Single	Multi	

All patients consented to donation and gave written, informed, consent to the use of tissue samples at the time of enrolment into the ICON 5 study which had appropriate regulatory and ethical approval (London Research Ethics Committee; MREC/02/2/3) (clinical trials.gov identifier NCT00011986).

### Immunohistochemistry

Each tumour sample underwent immunohistochemical staining for POSTN (Anti- POSTN 1.25μg/mL Oxford biosystems (RD18104050)), CXCL 14 (Anti- CXCL 14 2.5μg/mL Abcam, Cambridge, UK ab46010) and pSmad 2/3 (Anti- phosphor- Smad2, cell signalling Tech (3108S)) via the Bond-III automated IHC stainer following antibody concentration optimisation via hand staining. Deparaffinised sections were subjected to antigen retrieval (citrate buffer, pH = 6, in microwave for 2x5 mins), incubated with each primary antibody overnight at 4°C, visualised with a three-layer avidin-biotin technique and 3,3’ -diaminobenzidine, and counterstained with Mayer’s hematoxylin. Slides were scanned and images captured using a Leica SCN 400.

### Scoring

For the purposes of validation, methods and statistical analysis described by Reister *et al* were replicated exactly, taking details from the original paper and contacting the authors for clarification where necessary [14]. Each slide was scored in three separate 1mm^2^ pre-determined areas by two independent scorers (AH and MP), using QuPath– 0.2.0 –m8 software (Queens University Belfast, N.I.) at 20 times magnification. Each region was given a score based on the difference of staining intensity between tumour and stroma of 1, 2, or 3 (mild, moderate and strong respectively), multiplied by the percentage of tumour cells within that region displaying this staining intensity, represented as a score of 0 to 4 (<5%, 5–25%, 26–75%, and >75% respectively) Both scorers were blinded to clinical outcomes. Discrepancies in scoring were resolved by a third party (GW).

All slide scoring data was collated in MS Excel. Inter-scorer variability was determined using Spearman’s Rank Coefficient, and differences in cohorts when data were not paired was determined using Mann-Whitney-U test, both performed using Graphpad Prism version 8.4.3 (471). proteins predictive value, and combined score value was firstly calculated via simple logistic regression. Having confirmed strong inter-scorer correlation the scores from scorer one were used to generate the multivariable prediction model, created using logistic regression in WEKA, an open source machine learning software [18]. The dataset used can be found in supplementary materials. Creation of receiver operator characteristic curves (ROC curves) in Graphpad Prism version 8.4.3 (GraphPad Software, San Diego, CA). The discrimination and calibration performance of the model was determined by reporting AUC of ROC curves and p value. The code originally used by Riester *et al* in R was available from supplementary materials and was re-run on the validation dataset to ensure consistency. A p value of <0.05 was used to determine significance for all statistics.

## Results

**Fig 1:**
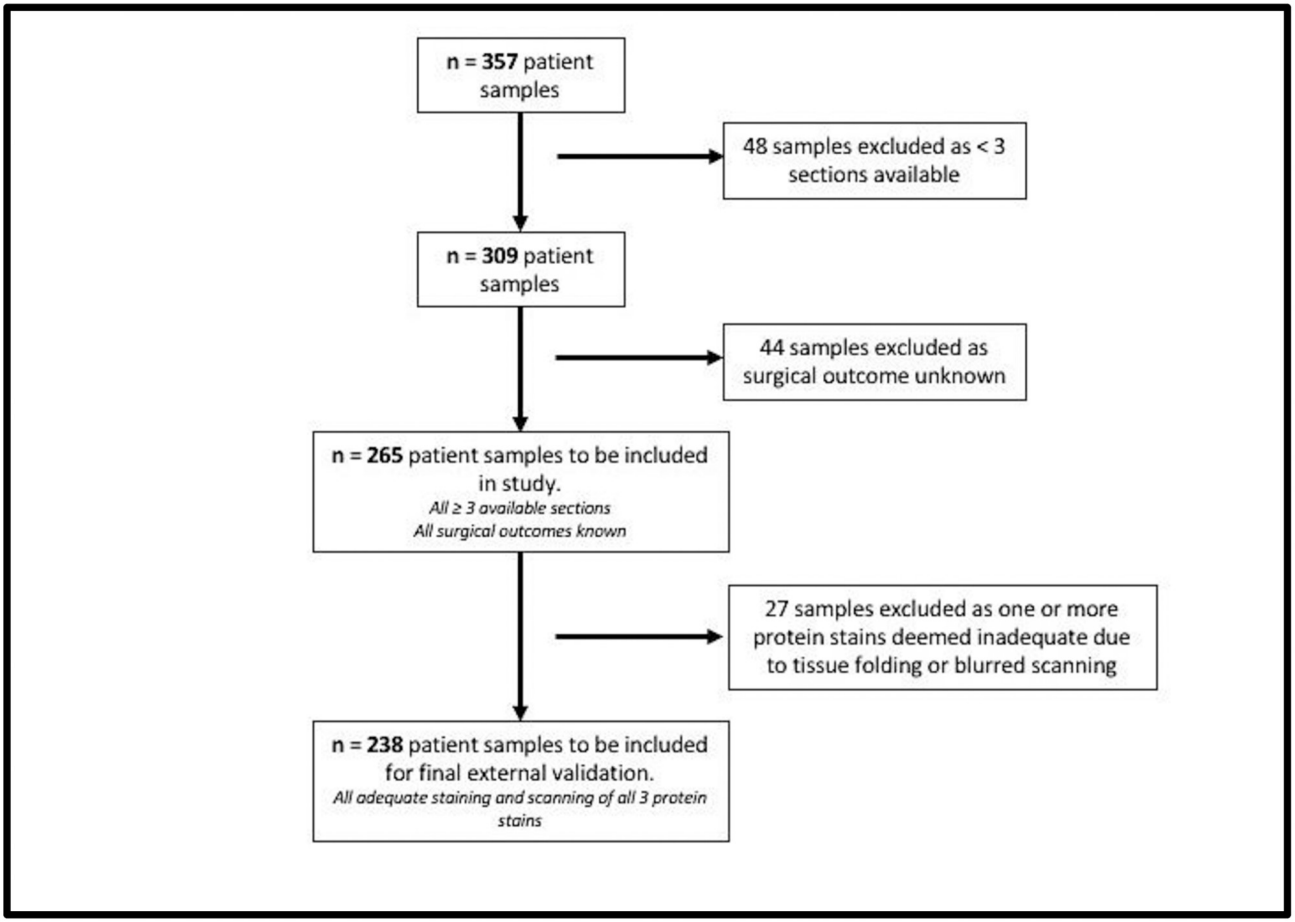
Consort diagram describing tumour sample selection process for inclusion in study.

238 patient samples were identified from the ICON5 clinical trial. All samples comprised a block or > = 3 formalin fixed paraffin embedded slides, Fig 1.

**Fig 2:**
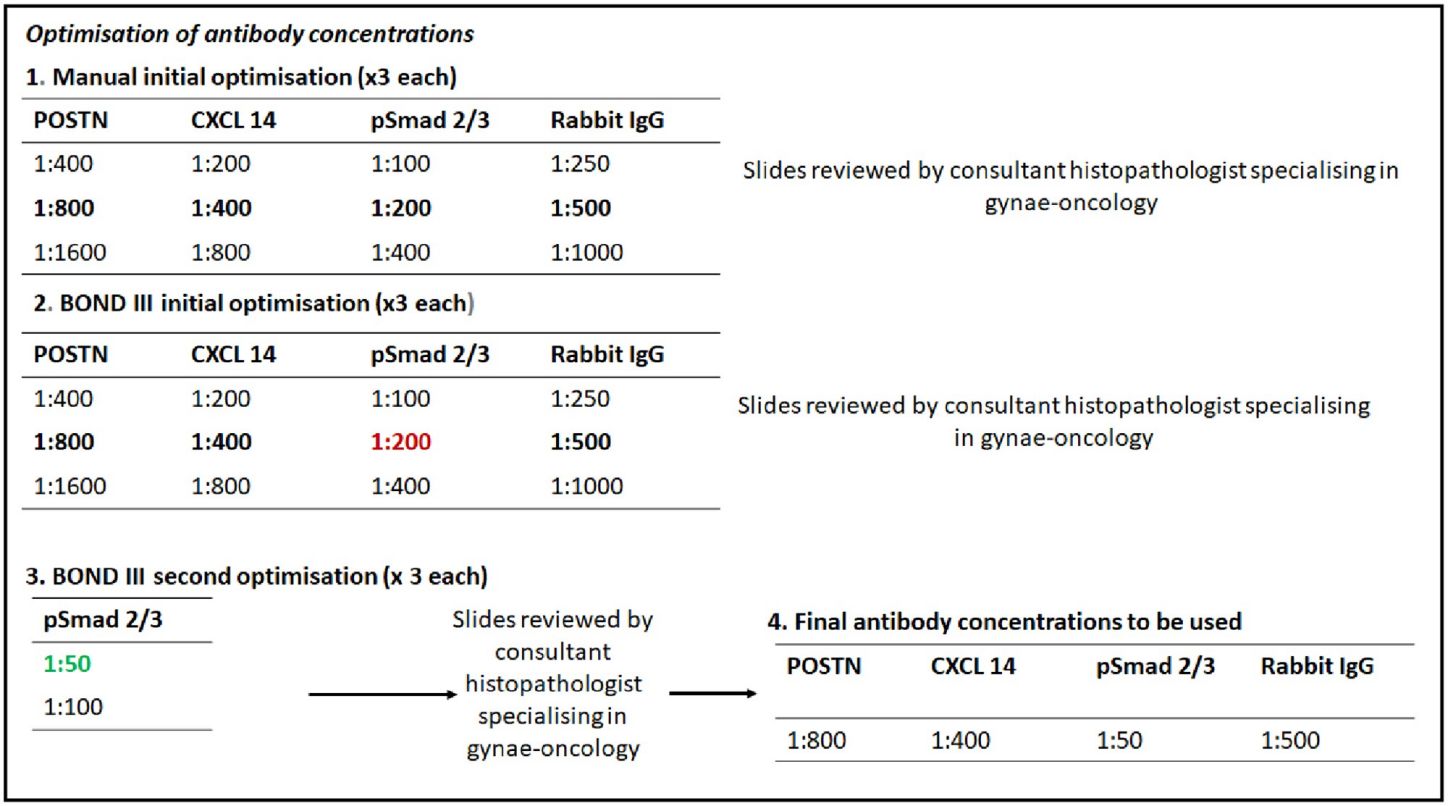
Optimisation process for antibody selection. Step one shows in bold antibody dilutionsused in original study. Three slides per dilution were stained for concentrations more and less dilute than the original. Following hand staining, stained slides were reviewed by the author and a consultant histopatologist with a specialty in gynae oncology, to ensure adequate staining. Step two again describes the range of dilutions stained on the automated platform. Review agreed adequate staining for POSTN and CXCL 14, however psMAD 2/3 appeared under-stained at dilutions used in the original paper. For this reason a further optimisation step was performed and a dilution of 1:50 was then agreed to result in adequate staining. Step four highlights antibody concentrations used in final IHC of whole validation cohort.

Antibody concentrations were determined by hand staining and confirmed using the automated staining platform, Fig 2. Antibodies for POSTN and CXCL 14 produced adequate staining at the same concentrations used in the original study (1:800, 1:400 respectively), however Anti- pSmad 2/3 was required at a more concentrated dilution (1:50) in order to achieve adequate staining levels.

Of the 238 patient samples included for analysis, all were from a high-grade epithelial subtype and originated from FIGO stage III and IV tumours. 202/238 (85%) patients underwent primary debulking surgery, with a suboptimal rate of 47%, a higher percentage than reported for the whole trial cohort (30%). Thus the number of events (suboptimal cytoreduction) was 112 for this validation cohort, Table 1.

Slides were scored as per the methods section above. The scoring for each protein was assessed, and all three showed a strong positive association between the two scorers (p < 0.001), Table 2. When a sum of the scores was calculated for each of the three proteins, again a strong positive correlation was demonstrated between the two scorers (R^2^ = 0.8025, 95% CI 0.7506–0.8445, p < 0.0001).

**Table 2:** Correlations between scorer 1 and scorer 2 for each protein expression assay.

	R^2^ (95% CI)	p
POSTN	0.749 (0.68–0.80)	<0.001
CXCL 14	0.700 (0.62–0.76)	<0.001
pSmad 2/3	0.836 (0.79–0.87)	<0.0001

**Fig 3:**
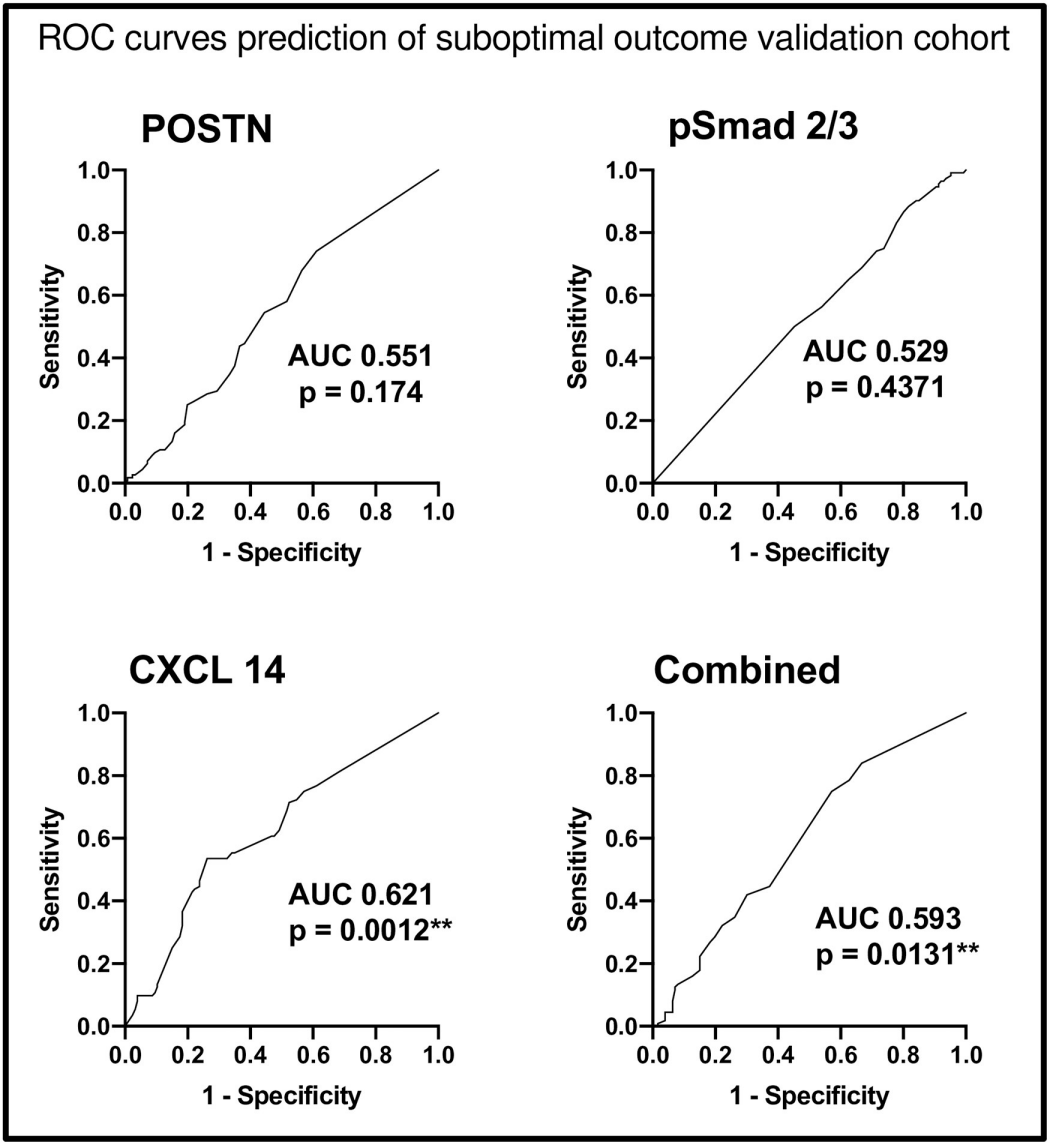
Receiver operating characteristics curves demonstrating poor predictive affinity for suboptimal debulking rates in the validation cohort, n = 238.

Logistic regression was utilised to externally validate four models (three individual protein models and one combined score model) in the total cohort of 238 cases, firstly taking each protein in turn and finally combining the individual proteins scores to create a combined score, Table 3. All four models were associated with poor performance to predict suboptimal cytoreduction (POSTN AUC 0.55 p = 0.174, pSmad2/3 AUC 0.53 p = 0.437, CXCL 14 AUC 0.62 p = 0.0012, combined score AUC 0.59 p = 0.0131), Fig 3.

**Table 3:** Comparison of median scores for each protein.

Cohorts		Original dataset Median score (IQ range)	Validation dataset Median score (IQ range)	p value
Complete cohort	CXCL 14	5 (4–7)	8 (5–12)	<0.0001*
	PSMAD 2/3	5 (3–7)	0 (0–2)	<0.0001*
	POSTN	4 (1–7)	1 (0–3)	<0.0001*
	Combined score	15 (9–19)	11 (7–14)	<0.0001*
Suboptimal cytoreduction	CXCL 14	7 (6–9)	7 (4–10.75)	0.548
	PSMAD 2/3	7 (6–8)	0 (0–2)	<0.0001*
	POSTN	8 (6–10)	1 (0–4)	<0.0001*
	Combined score	23 (17–26)	11 (7–13)	<0.0001*
Optimal cytoreduction	CXCL 14	5 (3–7)	9 (7–12)	<0.0001*
	PSMAD 2/3	5 (3–6)	0 (0–2)	<0.0001*
	POSTN	3 (1–5)	1 (0–3)	<0.0001*
	Combined score	12 (8–16)	12 (8–16)	0.557

p values calculated by Mann-Whitney U test, p <0.05 considered significant

As the original model contained only primary tumours, and our validation cohort contained 85% primary tumours (n = 202) and 15% tumours taken at the time of interval debulking surgery (n = 36), the analysis was repeated limiting to PDS samples only. Excluding the 36 IDS tumours from the analysis, there was marginal improvement in predictive ability for the four models (POSTN AUC 0.56 pSmad 2/3 AUC 0.54, CXCL 14 AUC 0.63, combined scores AUC 0.58), Fig 3.

A comparison of these results, alongside results from the original study are shown in Table 4.

**Table 4:** Comparison of AUC between each study cohort and for each model.

model	Area under curve (AUC)		
	Reister et al	All cases (n = 238)	Limited to PDS (n = 202)
POSTN	0.81	0.55	0.56
CXCL 14	0.79	0.62	0.63
pSmad 2/3	0.79	0.53	0.54
Combined model	0.87	0.59	0.58

## Discussion

Despite showing early promise on an internal validation cohort, the predictive affinity of this three protein signature is not replicated when applied to an external cohort of patients. This failure of validation may be attributed to overfitting of the original model and/or differing surgical practice between centres.

The accuracy levels achieved (AUC ≤ 0.621) for all models in this validation are scarcely more than chance, and therefore would not be acceptable for use in clinical practice.

The external validation was performed with care to ensure the techniques used to create the original model were replicated as closely as possible in the validation set. The conditions in which the IHC were undertaken were as similar as possible with the exception that the original model used hand staining, in contrast to this validation study which used automated staining. Despite this difference, hand staining was also successfully performed for the optimisation of antibodies in the validation model and all other materials and methods were kept consistent. Although the concentrations of the antibody anti-pSmad 2/3 did differ between the two studies, the working concentrations of anti-pSmad 2/3 used in the original study were not known. Differences may be accounted for by batch inconsistency.

There was very strong positive association between the two scorers in the validation cohort for all protein stains, which gives confidence in the consistency of the scoring.

Both studies used historical slides that had been stored between 4–20 years before IHC was undertaken. The validation cohort were stored at room temperature in a pre-cut paraffin fixed state. The method of storing used in the original study is not known. There are very few studies exploring the relationship between the time fixed slides are stored and the accuracy of IHC results. Some studies have suggested that longer storage time may be detrimental to antigenicity in tumour samples, resulting in false negative findings [19]. Conversely, other studies have contradicted this thinking, with Forse et al reporting adequate staining of breast cancer tissue via IHC following 12 years of storage [20], although these slides were stored at -80°C and not at room temperature. Consensus does agree however, that if slides are to be stored over prolonged periods, they must be paraffin fixed, as they were in this validation study. Both studies also included successful negative control, suggesting that a positive result was indeed a true positive. Staining was also reviewed by an experienced consultant histopathologist (GW), who confirmed that despite their age, the slides have stained adequately.

Many previously published surgical prediction models have also failed to successfully validate when applied to external cohorts, and this is often attributed to the differences between the cohorts, with this study being no exception. Most notably, the original study cohort underwent surgery in a single institution, whereas the validation cohort were made up of patients from multiple different centres internationally. Variation in surgical practice within centres is well established, and surgeon heterogeneity between centres is vast [21–23]. This variation in practice may explain some of the differences seen between the two cohorts. However, the ICON5 study recruited patients from many centres in the UK and thus more likely represents clinical practice.

This external validation was conducted with adequate power and replicated the methods and materials used in the previous internal validation. Despite this, the three-protein prediction model failed to accurately predict suboptimal surgical outcome in this cohort.

Furthermore, given the poor predictive accuracy seen here, it is unlikely that the addition of further cohorts would change this finding significantly.

Future work should therefore focus on identifying different biomarkers that may offer more accurate prediction for the outcome of surgery.

## Supporting information

S1 Dataset (XLSX)
